#  Distributor– Retailer Interface in Pharmaceutical Supply Chain: Service Quality Measurement Scale

**Published:** 2016

**Authors:** Gholamhossein Mehralian, Jafar Babapour, farzad peiravian

**Affiliations:** a *Department of Pharmacoeconomics and Pharma Management, School of Pharmacy, Shahid Beheshti University of Medical Sciences, Tehran, Iran.*

**Keywords:** Pharm supply chain, Service quality, Pharm, Iranian

## Abstract

In the current competitive market, service quality management is the key to the survival and success of businesses. SERVQUAL is a popular service quality measurement scale (SQMS) that has served as a basis for subsequent research on service quality; it has been used for testing different aspects of service quality in a market. The purpose of our study is, therefore, to develop a service quality measurement scale (SQMS) for the distributor–retailer interface of Pharm supply chains (PSC) in Iran. A survey was performed to collect data from pharmacies located in Tehran. A valid and reliable questionnaire delivered to pharmacies, and 400 pharmacies were intended to participate in our survey. Confirmatory factor analysis (CFA) was used to develop an SQMS in this study. Sufficient sampling was undertaken to do CFA. Consistent with other service quality studies, this Res developed an SQMS with five dimensions and 20 items for PSC, and contributes to mangers to regularly measure service quality. This is an initial study to develop a framework for measuring service quality in Iranian PCS. The framework can be used effectively to achieve competitive advantage at the distributor–retailer interface.

## Introduction

In the current competitive market, service quality management is important for the survival and success of businesses, and Mark studies demonstrate that high service quality is a critical factor in the competitive business world (1). It is needless to say that Companies do their best to improve their service quality and satisfy customers by delivering their products in an accurate and prompt manner (2). The concept and definition of service quality have been proposed by Parasuraman *et al.* (3, 4). The authors define service quality as the perceptual gap between expectations and evaluation of service experiences.

Generally, researchers are in favor of one of the two conceptualizations. The first model that has clarified the dimensions of service quality as functional and technical quality is the “Nordic perspective”. The second one uses terms that explain service encounter characteristics; this is called the “American perspective.” Although the American perspective dominates the literature, the consensus about the relative value of these two approaches has not been reached (5).

There are many studies trying to measure the service quality; however, it is not clear what values should be measured (6). The main problem is the intangibility of services, which makes it difficult to identify, measure, and manage the crucial service aspects. There are a variety of definitions of service quality in the literature. On the basis of these definitions, several different measurement scales have been developed. Among these, SERVQUAL and SERVPERF are the two most popular measurement scales for service quality (7).

Since improvements in service quality can increase the overall success rate of the healthcare provision, they have become an important topic to healthcare providers and marketers and the focus of many studies. Service quality can also substantially affect patient satisfaction (8). Inevitably, the increased satisfaction improves customer loyalty and purchase intention (9, 10). Accurate measurement of the quality of healthcare services and identification of the nature of service delivery system are equally important for the success of healthcare providers (11). Many researchers and practitioners are interested in studying service quality because it directly affects business performance, customer loyalty, profitability, and customer satisfaction (12, 13).

Most of the Res on service quality has been focused on the end user (14). The interactions between different parts of the PSC (as an integral part of healthcare services) have been rarely studied. These interactions are important; the services received by patients are strongly affected by the service quality of these supply chains, including the distributor–retailer interface. Here we developed a service quality scale for the distributor–retailer interface of PSC in Iran. Our results should at least partly ameliorate the lack of data on the service quality in supply chains. The paper also discusses developing an SQMS in an important but neglected sector of distributor–retailer interface of PSC. SQMS described here will help the managers of Iranianian Pharm distribution companies to measure, manage, and improve the service quality. The rest of paper are organized accordingly; in Section 2 a literature review is presented, followed by the methodology in Section 3. In Section 4, the study results are organized and in Sections 5 and 6, the discussion and conclusion of study are provided.

*Literature review*

*Service quality definition*

The measure of quality is the ability of a product or service to fulfill the requirements or perform the task for which it is designed. Unlike a product, a service is judged not only by the outcome (technical quality)‚ the process of delivering a service is also assessed to evaluate its quality (functional quality) (15, 16). Different definitions have been offered for service quality and various measurement scales have been developed accordingly (7). Service quality can be defined as the difference between customer expectations and the perceived service performance. When the performance is lower than the expectations, the quality offered is not sufficient and consequently the customers become dissatisfied (17).

According to Boulding *et al.* expectations are “pre-trial beliefs about a product or service” (18). In other words, service quality is a universal attitude or judgment about the level of the service. The early studies for measuring service quality are based on the Res in the goods sector. Parasuraman *et al.* examined this area in the mid-1980s (3). They have been among the first researchers to conclude that the nature of quality in the service sector is different from that in product sector. Brady and Cronin, and McAlexander *et al.* have applied the term “technical care” in evaluation of the outcome of healthcare services and concluded that it is a prominent factor in patient perception of service quality (5, 10). From another perspective, de Ruyter and Wetzels have also reported that the service quality and service outcome are directly related (19). To summarize, identifying, and measuring the service quality is difficult as the concept may be discussed from different points of view (20).

*The measurement of service quality*

Edvardsen *et al.* state that analysis and measurement are the basis for developing service quality (21). To make a system operate effectively, we need an instrument for identifying and measuring its quality. The specific characteristics of services mainly their intangibility, inseparability, perishability, and incongruity make the measurements difficult (22). Based on definition proposed by Parasuraman *et al.* service quality is defined as the difference between the expectations of customers (“what they want”) and their perceptions (“what they receive”) (3, 4). Accordingly, they have proposed a SERVQUAL scale for the service quality measurement. The SERVQUAL scale has been a turning point in the history of service quality assessments; it is now widely applied.

*Service quality models*

The perceptions of service quality are based on a variety of dimensions. However, there is no general agreement on nature or number of dimensions. Two, three, five, and even ten dimensions have been proposed. The assessment of service quality is a highly complicated process that may involve several levels of abstraction (5). Several models of service quality have been proposed (23).

In this section, the top five of 

Service Quality Measurement models are described.

- The Nordic Model
- Nordic model is based on disconfirmation paradigm, which compares the perceived performance with expected service (24). Grönroos has proposed that consumer perception of service quality is a function of a gap between their expectations and perception of the quality of the delivered service (16). This conceptualization of service quality has provided the framework for the SERVQUAL scale developed by Parasuraman *et al.* (4).
- The SERVQUAL Model
- Parasuraman *et al.* have developed a new model of service quality measurement based on disconfirmation paradigm (3). They have tried to overcome the weaknesses of the Nordic model by offering a new way to measure the service quality. In their SERVQUAL model, the gap or difference between expected and delivered service is used to measure the service quality. The model uses five dimensions: reliability, responsiveness, assurance, empathy, and tangibility. They have declared that the five dimensions and 22 factors proposed in their American perspective are non-exclusive in nature and applicable to all service firms (4).
- The Three-component Model
- Rust and Oliver offer a three-component model: the service product (i.e., technical quality), the service delivery (i.e., functional quality), and the service environment are considered (25).
- The Multilevel Model
- To compensate for the inconsistencies reported for SERVQUAL factors, Dabholkar *et al.* have proposed a multilevel model of service quality (26). They have changed the model structure to a three-stage model, including overall perceptions of service quality, primary dimensions, and sub-dimensions. This model has been defined for the evaluation of service quality in retail stores. Although this multilevel model offers a useful new structure, it needs to be generalized. They have proposed five dimensions central to the service quality: physical aspects, reliability, personal interaction, problem-solving, and policy.
- The Hierarchical Model
- In 2001, Brady and Cronin constructed a new model by combining other models; they improved SERVQUAL by defining what items needed to be reliable, responsive, empathic, assured, and tangible. They assumed that the perception of service quality would be based on customer evaluations in three dimensions: interaction quality (i.e., functional quality), physical environment quality, and outcome quality (i.e., technical quality) (5).
- During recent decades, the Researchers have attempted to develop a perfect model for measuring service quality, covering all the factors and coping with the problems in this area. The present study also addresses some of the important issues in the field. To achieve this goal, we selected the SERVQUAL model as the theoretical framework.

*SERVQUAL model*

The SERVQUAL scale has served as a basis for Res on service quality; it has been used for testing different issues related to service quality in the market. Parasuraman *et al.* have developed a 34-item service quality scale consisting of ten dimensions (reliability, responsiveness, competence, access, courtesy, communication, credibility, security, understanding/knowledge of the customer, and tangibles) (3). Their later work has delivered an SQMS with 22-items and five dimensions (4):

(1) Tangibility. Physical facilities, equipment, and appearance of the personnel.

(2) Reliability. Ability to perform the promised service dependably and accurately.

(3) Responsiveness. Willingness to help customers and provide prompt service.

(4) Assurance. Knowledge and courtesy of the employees and their ability to inspire trust and confidence.

(5) Empathy. Caring, individualized attention provided by the company.

The accuracy of conceptualizing the SERVQUAL scale as consisting of the five distinct components identified by Parasuraman *et al*. has been questioned (4). However, the validity of the 22 individual performance scale items that make up the SERVQUAL scale appears to be well supported both by the procedures used to develop the items and by their subsequent use. Furthermore, some studies have found that the numbers of service quality dimensions are not stable across different services, using factor analysis (27, 28, 11). There seems to be a consensus that SERVQUAL is not a generic solution for all service industries, and other service-specific dimensions have been suggested (29). The construct validity of SERVQUAL needs to be tested on industry-by-industry basis before it can be used to test the customer perception of service quality.

*Retail service quality scale*

Finn and Lamb state that in the retail industry, the SERVQUAL scales are not suitable for measuring the five constructs identified by Parasuraman *et al.* They have tested SERVQUAL in four different types of retail stores using confirmatory factor analysis. They have not found a good fit to the proposed five-factor structure and concluded that unmodified SERVQUAL should not be used as a valid measure of service quality in a retail setting. However, they have not proposed any alternative acceptable structures or scales (20). Thus, SERVQUAL should not be considered an “off the shelf” measure of perceived service quality. Mattsson has found that the SERVQUAL scale of retailing is applicable for businesses offering more goods than services, like supermarkets. However, for the retailing contexts where the services are more prominent than the goods (e.g., electronics stores) SERVPERF is more appropriate (30).

*Service quality in supply chain*

In spite of the universal recognition of the importance of service quality in supply chains, it has not been studied adequately. Several researchers have attempted to develop a conceptual model of service quality in a supply chain context (14). It is clear that the majority of Res on the service quality have focused on the consumer (12). The lack of studies of the service quality in supply chains is noticeable. However, a variety of performance measures has been used to study the performance of supply chain systems. The SERVQUAL model differs from other scales since it applies the terms that describe determinants of service perception. Therefore, the five dimensions of SERVQUAL might be used to refine some aspects of service quality (5).

The Pharm industry uses many processes, operations, and management procedures involved in the discovery, development, and manufacture of drugs and medications. The PSCs are the paths through which the essential Pharms should be distributed to the consumer at the right quality, right place, and the right time (31). The PSCs are very complex and responsive to ensure the delivery of medicines to the patients at the right time and under the right circumstances, to cure diseases or alleviate suffering. 

This is a very sensitive supply chain, in which even minor errors are unacceptable because of their direct impact on health and safety. Today, the Pharm industry is faced with many challenges. There is an ever-increasing competition, especially in the generics industry, and the need to reduce time-to-market periods. There are also constant demands to increase Res and development productivity and decrease the Pharm life cycles. 

**Figure1:**
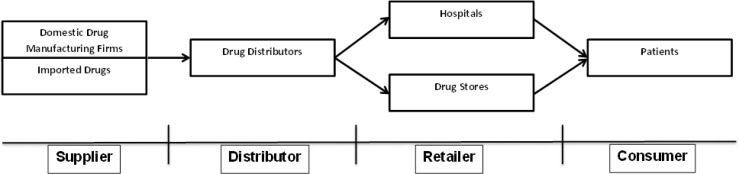
Pharm supply chain

The companies have to face regulatory obstacles and barriers to entry into the global market, maintain production flexibility and decrease production costs (32). Figure 1 shows a simple model of a PSC. A PSC is made up of different members, including manufacturers, secondary producers, market warehouse/distribution centers, wholesalers, retailers/hospitals, and patients (33).

*Methodology*

*Developing the SQMS*

The five-dimension SQMS proposed by Parasuraman is the most frequently used method for measuring the service quality. It has been reported that this scale can be used to measure service quality in different areas, including supply chains (34) and hospital environment (35). Although it has been successfully used by Niaz Ahmad *et al*. in Pharm distribution sector (14), we decided to use this SQMS to measure the satisfaction level in the Iranian pharmacies that they are practicing in a supply chain at which the regulations and socioeconomic variables are entirely different from that of the mentioned study.

*Refinement of the questionnaire*

Parasuraman’s SQMS includes five dimensions with 22 items. For the purpose of this Res, we held an expert panel with Pharm experts, sale managers, and purchasing agents. A questionnaire including 5 dimensions and 28 items was developed in Persian. Next the questionnaire was presented to 15 distributor–retailer experts to test the content and face validity of the modified SQMS. The comments of the participants were received within 5 working days. 

The questionnaire was finalized after slight modification of some of the questions. It was based on a 5-point scale (1 for completely unimportant and 5 for very important). The questionnaire is presented in Table 1.

*Data gathering*

There are about 8500 pharmacies in Iranian and 24% of them (2000) are located in Tehran (36). Around 40 main distributors deliver medicines to the pharmacies in the country via their agencies. The distributors are allowed to provide services to any pharmacies and the pharmacies are free to choose the best service providers. In recent years, increasing numbers of distributors have highlighted the importance of competitive advantage and high-quality services. A suitable SQMS model for Iranianian Pharm industry is urgently needed, particularly for evaluation of distributors. Measuring the quality of current services in distributor–Pharm interface is also of great importance. We distributed 2050 questionnaires to pharmacies located in Tehran, and 400 were returned (response rate of 19.5%). The issue of response rate is a not critical matter, but rather it is so important a study to reach the number of returned questionnaires which are enough good to be representative a population for further analysis.

The details of respondent profiles are provided in Table 2.

*Data analysis*

*Scale purification and statistical analysis*

For assessing construct validity and reliability of dimensions and their related items, factor analysis and Cronbach’s alpha were used, respectively. The following results were obtained:

- Reliability: This dimension included 8 items of which three were deleted (Rel6, Rel7, and Rel8); loading factors of remaining items were from 0.601 to 0.766. The average variance extraction (AVE) of the items was 50%. Cronbach’s coefficient alpha for this dimension was 0.739.
- Tangibility: This dimension included 5 items of which one of was deleted (Tan1) using confirmatory factor analysis (CFA). The factor loadings of the remaining items ranged from 0.683 to 0.768. The AVE of the items was 52%. Cronbach’s coefficient alpha for this dimension was 0.69.
- Assurance: This dimension included 7 items of which three were deleted (Ass1, Ass4, and Ass5); loading factors of four remaining items were from 0.658 to 0.788. AVE of these four items was 51%. Cronbach’s alpha coefficient for this dimension was 0.680.
- Empathy: This dimension included 5 items, one of which was deleted using CFA (Emp4). The factor loadings of the remaining items ranged from 0.663 to 0.861. The AVE of the items was 54%. Cronbach’s alpha coefficient for this dimension was 0.711.
- Responsiveness: This dimension included 3 items; none was deleted after CFA. The factor loadings of the related items ranged from 0.808 to 0.868. The AVE of the items was 70%. Cronbach’s alpha coefficient for this dimension was 0.782.

**Figure 2:**
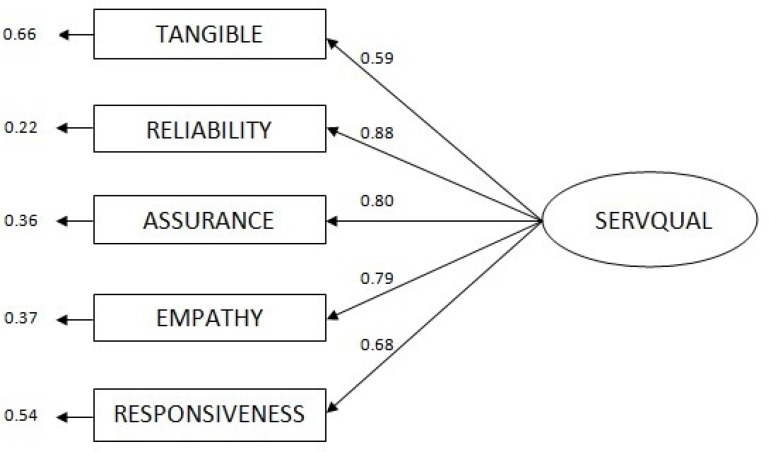
Structured model of SQMS

As we have mentioned earlier, the measurement instrument selected for this study is based on Parasuraman’s model (4), modified after focus group discussions. Therefore, the content and face validity were verified.As Table 3. shows, all factor-loading values are higher than 0.6 and AVE values are higher than 50%; accordingly, convergent validity of the construct is confirmed. Furthermore, the value of the AVE for each dimension is more than squared value of the correlation between dimensions, demonstrating discriminant validity.Goodness for fit assessment of the overall model was conducted using LISREL 8.52 statistical package to capture the specified causal relationships in the purified and localized SQMS. These relationships, with associated statistical values, are illustrated in Figure 2.Table 4. shows absolute fit indicators, incremental fit indicators, and goodness-of-fit index (GFI). All the fit indices are within the acceptable ranges (37-40).

## Discussion

This is the first study conducted to develop an SQMS in Iranian PSC context. We obtained a reliable and valid measurement scale for measuring the service quality of the distributor–Pharm interface of PCS. We developed a measurement scale consisting of 5 dimensions, based on the previously published schemes (3, 4, 14). This scale is useful for assessing service quality of the distributor-pharmacy interface and can be used to improve the service quality and customer satisfaction. For the Pharm distributors, it is a suitable scale to achieve competitive advantage by analyzing the customer data.

**Table 1:** Questionnaire items

**Deleted or Remained** **item after** ** factor analysis**	**Item**	**Question** **number**
**Reliability**		
Remained	Temperature and humidity are controlled during transportation of drugs	**Rel1**
Remained	When you have any problem, distributor shows a sincere interest in solving it	**Rel2**
Remained	Shipments contain correct items	**Rel3**
Remained	Shipments contain incorrupt items	**Rel4**
Remained	Shipments contain incorrect quantity	**Rel5**
Deleted	Records are kept confidential	**Rel6**
Deleted	Payment information is kept confidential	**Rel7**
Deleted	Distributor provides legal support when needed	**Rel8**
**Tangible**		
Deleted	Distribution center has modern equipment (computers, air-conditioning, etc.)	**Tan1**
Remained	Distributor has sufficient physical facilities for storing drug products	**Tan2**
Remained	The physical facilities at distribution center are visually clean	**Tan3**
Remained	Vehicles used in transportation are visually in a good condition	**Tan4**
Remained	Personnel handling drugs are professional in appearance	**Tan5**
**Assurance**		
Deleted	Personnel at the distribution center are trained	**Ass1**
Remained	Order taking methods (including frequency) are accurate	**Ass2**
Remained	Order delivery methods (including frequency) are accurate	**Ass3**
Deleted	Personnel in the distribution center are consistently courteous with you	**Ass4**
Deleted	Personnel in the distribution center have the knowledge to answer your queries	**Ass5**
Remained	Personnel in the distribution center have the authority to solve your problems	**Ass6**
Remained	The distributors are consistently eager to provide you services	**Ass7**
	**Empathy**	
Remained	Distribution personnel fulfill your emergency orders	**Emp1**
Remained	Distribution center has office working hours suitable to you	**Emp2**
Remained	Distribution center has staff working hours suitable to you	**Emp3**
Deleted	Methods designed for payments are convenient to you	**Emp4**
Remained	Distribution center personnel’s fulfills your specific requirements	**Emp5**
	**Responsiveness**	
Remained	Distributor responds immediately to your enquiries	**Res1**
Remained	Distributor responds immediately to your complaints	**Res2**
Remained	When distributor promises to deliver by certain time, they do so	**Res3**

**Table 2:** Demographic profile of respondents

Variable	Frequency (N)	Percent (%)
Age		
25-34 35-44 45-54 55-64 65< Total	47 137 146 58 12 400	11.8 34.2 36.5 14.5 3.0 100
Gender		
Male Female Total	225 175 400	56.2 43.8 100
Working precedent		
<10 year 11-20year 21-30year >30year Total	133 123 128 16 400	33.2 30.8 32 4 100

**Table 3:** Factor analysis results

**Dimension**	**Mean**	**No. of deleted items**	**KMO**	**Average variance** **extracted (AVE) %**	**Factor Loadings**	**Cronbach’s coefficient (α)**
Reliability	4.5	3	0.791	50	0.601-0.766	0.739
Tangible	4.2	1	0.736	52	0.683- 0.768	0.695
Assurance	4.3	3	0.653	51	0.658-0.788	0.680
Empathy	4.4	1	0.685	54	0.663-0.861	0.711
Responsiveness	4.5	0	0.694	70	0.808-0.868	0.782

**Table 4:** Goodness-of-fit measures

**Fitness indicator**	**Suggested criteria**	**Validation value**
*Absolute fit indicators*		
c2/df GFI RMR RMSEA	<3 >0.90 <0.05 <0.08	2.8 0.93 0.04 0.06
*Incremental fit indicators*		
AGF NFI CFI IFI	>0.90 >0.90 >0.90 >0.90	0.95 0.96 0.97 0.94
*Goodness of fit index*		
PNFI PGFI	>0.5 >0.5	0.6 0.7

LISREL results show that reliability dimension has the strongest effect on SQMS. This implies that the reliability is perceived to be the most important factor affecting the satisfaction of pharmacies receiving services from Pharm distributors. Considering the sub-items of the reliability, it can be argued that the Pharm managers are highly affected by the behavior of distributors such as their commitment to the qualitative and quantitative accuracy of deliveries and availability of the requested products. We believe that implementation of online ordering system can be a breakthrough for PSC with many benefits for both suppliers and buyers. Reports from other countries indicate that the role of government in enforcing that policy is greatly appreciated (41, 42).

According to the views of respondents, assurance was the second most important factor. The Pharm suppliers should select and train motivated people, to satisfy their customers by delivering precise and regular services. Their staff also should be competent enough to solve everyday problems.

In the current competitive market in PSCs, empathy with customers is of great importance; without it, a good interaction cannot be achieved. Indeed, the Pharm suppliers should try to establish the atmosphere of mutual understanding and trust. The next two factors affecting SQMS values were responsiveness and tangibility. The latter factor is associated with items, which are more tangible for pharmacies employees in comparison with others. Therefore, the suppliers should use neat and clean equipment, visibly in good condition.

This study confirmed the validity of the five dimensions of Parasuraman *et al.* using CFA (3, 4). However, our findings are not in line with the recently published study of Niaz Ahmad *et al.* who approved just four dimensions of the original work and excluded many items (14). In the context of PSC, this difference is not unexpected; it can be explained by the socioeconomic diversity of different nations.

## Conclusions and limitations

As in other industries, Pharm companies are in intense competition with their rivals; an important part of this competition takes place in PSCs where Pharm distributors try to increase their market share by capturing the business of pharmacies. The satisfaction of pharmacies is critical for such companies. Our study contributes to the development of a valid and reliable framework for evaluation of the quality of services received by the pharmacies. Such framework can help the managers to make informed business decisions. The data for this study were collected from retailers in Tehran. Hence, service quality measurements for the retailers in cities other than Tehran may not necessarily follow the same pattern.
